# Analytic Complexity and Challenges in Identifying Mixtures of Exposures Associated with Phenotypes in the Exposome Era

**DOI:** 10.1007/s40471-017-0100-5

**Published:** 2017-01-18

**Authors:** Chirag J. Patel

**Affiliations:** Department of Biomedical Informatics, Harvard Medical School, 10 Shattuck St, Boston, MA 02115 USA

**Keywords:** Mixtures, Machine learning, Mixtures, Combinations, Exposome

## Abstract

**Purpose of Review:**

Mixtures, or combinations and interactions between multiple environmental exposures, are hypothesized to be causally linked with disease and health-related phenotypes. Established and emerging molecular measurement technologies to assay the *exposome*, the comprehensive battery of exposures encountered from birth to death, promise a new way of identifying mixtures in disease in the epidemiological setting. In this opinion, we describe the analytic complexity and challenges in identifying mixtures associated with phenotype and disease.

**Recent Findings:**

Existing and emerging machine-learning methods and data analytic approaches (e.g., “environment-wide association studies” [EWASs]), as well as large cohorts may enhance possibilities to identify mixtures of correlated exposures associated with phenotypes; however, the analytic complexity of identifying mixtures is immense.

**Summary:**

If the exposome concept is realized, new analytical methods and large sample sizes will be required to ascertain how mixtures are associated with disease. The author recommends documenting prevalent correlated exposures and replicated main effects prior to identifying mixtures.

## Introduction

We are exposed to many different factors simultaneously throughout our lifespan, ranging from drugs, infectious agents, environmental pollutants, and macro- and micronutrients. The *exposome* is an emerging attempt to conceptualize and characterize the breadth of exposures humans encounter from birth to death [1•, 2•]. The exposome concept promises to capitalize on our accelerating ability to measure indicators of environmental exposure in a high-throughput manner leveraging new tools, such as metabolomics [3]. In fact, investigations are now underway to measure the exposome in children to discover new associations in development-related traits, such as the National Institute of Environmental Health Sciences Children’s Health Exposure Analysis Resource (CHEAR, see https://www.niehs.nih.gov/research/supported/exposure/chear/). In contrast, most epidemiological investigations to date consider one or a few exposures at a time and we currently lack data-driven methods to associate, and discover, numerous environmental exposures, including mixtures, in etiological studies of disease.

New high-throughput measurements provide data to potentially identify mixtures, defined as a combinations of co-occurring exposures, associated with disease. Identification of mixtures is important for public health and epidemiology because it is hypothesized that exposures induce changes in phenotype (disease and health indicators) not as a single agent, but as a collection of agents acting in concert [4, 5]. In other words, mixtures can be further thought of as groups of exposures that together induce a change in phenotype that is different than the effect of each exposure component separately. We note that the search for mixtures may contrast with the well-known Bradford Hill criteria to assess causality [6]. Specifically, Hill’s criterion 3 is one of *specificity* or, in other words, the simpler the association between exposure and disease, the more likely the association is causal [6]. In the era of high-throughput measurement, Bradford Hill may need to be revisited if evidence for combinations of exposures associated with diseases are realized [7••, 8]).

First, we present some definitions. We define *phenotype* as a manifestation of a trait, such as disease and quantitative characteristics (e.g., height, body mass index). In environmental epidemiological investigations, we attempt to model the relationship between phenotypes (e.g., a disease or quantitative characteristic) and exposures. We define exposure *combinations* as a set of two or more exposures that occur together. Exposure co-occurrences are a pair of two exposures that co-occur together. Finally, *mixtures* are potentially interacting combinations of exposures that are associated with a phenotypic change. In other words, the potential effect on the phenotype is influenced not by the individual constituents of the mixture, but the combination. In the following sections, we aim to further define analytically both co-occurrences and mixtures of exposures and their associations with phenotype.

Analytic identification of mixtures of exposures in phenotype is fraught with challenges [9•, 10•]. In the following, we review existing and emerging analytic methods to understand the complex phenomena of co-occurring exposures and mixtures in the epidemiological setting and open challenges, extending the insights from Braun and others [11••].

## Potential Complexity of Identifying Mixtures: Expansive Number of Possible Combinations of Exposures and Mixtures

The total number of sets of exposures that can exist is immense. Suppose we have the capability of measuring *M* number of exposures in an epidemiological study. A combination of exposures can have any size: two (e.g., lead and cadmium), three (e.g., lead, cadmium, and bisphenol A), or more. In a setting with binary exposure variables (e.g., median split) and without any prior hypotheses, the total number of potential combination of high exposures that can occur in a dataset of size 2 and up to *N* is defined as “*M* choose *N*”, equal to m!/(n-m)!*m! where *M* is defined as the number of high exposures. For example, if our study had measured three (e.g., serum lead, cadmium, and arsenic) exposures in total, the number of possible combinations of size two (*M* = 2) is 3, including (lead, cadmium), (lead, arsenic), and (arsenic, cadmium). Suppose we measure 100 exposures (*N* = 100): the total number of combinations of size two that could co-occur is 4950. Of size 5, the total combinations are on the order of millions (exactly 75,287,520). One can imagine the scale of the number of combinations when scaling up to 100s of exposures of the exposome. Therefore, a primary challenge of identification of mixtures in phenotypes is the expansive number of exposure combinations possible.

## Dense Correlations and Examples of Co-occurring Exposures in NHANES

While there are a vast number of possible combinations of exposures, investigators may choose to focus on the most prevalent co-exposures or those that co-occur more frequently in the population. One way to identify co-occurring exposures includes estimating the correlation between each pair of exposures (e.g., through a Spearman or Pearson correlation measure) and “grouping” the highly correlated exposures with an “unsupervised machine learning” technique, such as clustering.

**Fig. 1:**
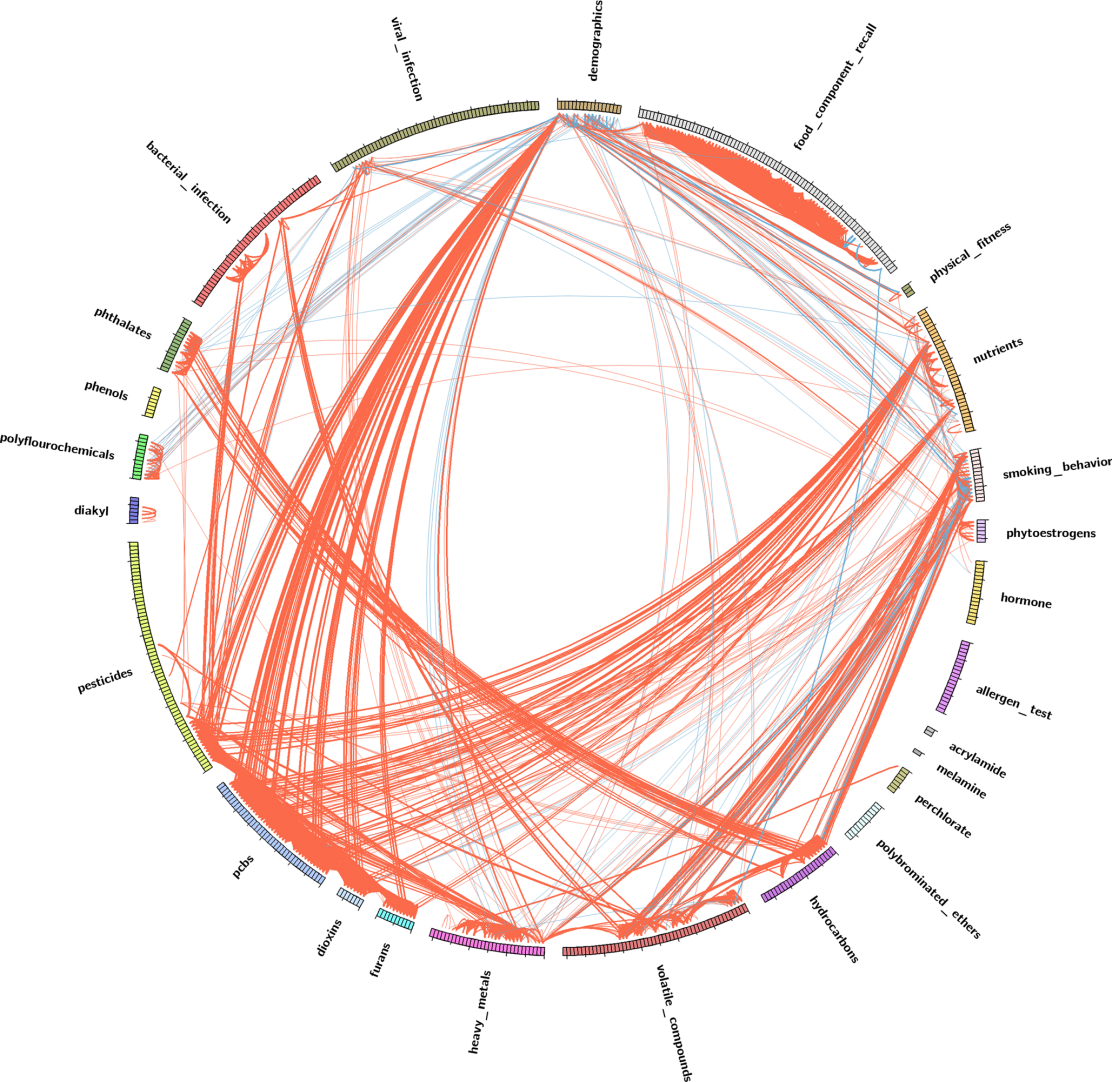
Visualization of an exposome globe (reproduced with permission from Patel and Manrai [12•]). Exposure measurements are arranged in groups in a circle. Pairs of exposures that are positively correlated with each other are linked and as *red edges*, while those negatively correlated are in *blue*. Shown are 2656 replicated correlations (or edges) in NHANES

Others and we have described how to identify prevalent co-exposures by displaying the correlation between multiple exposures and finding co-occurring exposure “networks” through unsupervised learning [12•] based on “relevance networks” [13]. Briefly, a network is composed of a combination of exposures, or nodes, whose connections are estimated by the correlation between exposures (e.g., Spearman or Pearson correlation). We assess the strength of the connection between exposures in a network by estimating the *p* value of significance through permutation-based tests. Specifically, each exposure is randomly permuted (sampled without replacement), and the correlations are re-computed to create a set of correlations to reflect a null distribution of no correlation. Then, we choose the most significant pairwise correlations to construct the network. Networks are then visualized in a “correlation globe” (Fig. 1), whereby each exposure variable is arranged in a circle (“nodes”) and lines between nodes indicate non-random correlation between exposure variables. There are many ways to create such visualization; for example, we created Fig. 1 with the Circos visualization package [14].

We implemented this method using epidemiological survey data from the National Health and Nutrition Examination Survey (NHANES), systematically computing pairwise correlations between 289 exposure variables, or 81,937 total correlations possible. Of these, the exposome network consisted of 2656 significant and replicated pairwise correlations. We identified several co-occurring exposures that are well known (see Table 1 for examples of exposures positively correlated with serum PCB170, cadmium, and β-carotene). The correlation structure is potentially “dense” (Fig. 1), and many exposures co-occur with many others [7••, 12•, 15, 16••]. Therefore, while the total number of combinations of exposures can be impossibly expansive as described in the previous section, the total number of prevalent co-occurring combinations of exposures can be reduced in magnitude but is still is a challenge to dissect.

**Table 1:** Examples of indicators of exposures positively correlated with serum beta carotene, cadmium, and PCB170 in the NHANES participant population, derived from [12•, 26••]

Beta carotene	Cadmium	PCB170
Alpha carotene	Lead	PCB172
cryptoxanthin	Cotinine	PCB177
Lutein and zeaxanthin	Hepatitis A titer	PCB178
Retinyl palmitate	Vitamin E	PCB180
	Cadmium (urine)	PCB183
	Blood toluene	Cadmium
	Blood m/p xylene	Lead
		Oxychlordane
		Vitamin E
		1,2,3,7,8-Pentachlorodibenzo-*p*-dioxin
		1,2,3,4,7,8-Hexachlorodibenzo-*p*-dioxin
		1,2,3,7,8,9-Hexachlorodibenzo-*p*-dioxin
		1,2,3,4,6,7,8-Heptachlororodibenzo-*p*-dioxin

All are serum measures unless indicated

We offer an additional cautionary note: by only focusing on combinations that co-occur frequently, investigators will lose opportunities to identify mixtures that involve dependencies between rare exposures. For example, it may be possible that hypothetical exposures *A* and *B* do *not* frequently co-occur; however, a phenotypic response may be induced in the rare chance that individuals encounter *A* and *B*. Or, put even more simply, there could be exposures whose influence on a phenotype is “triggered” by another exposure B. Correlation, or co-occurrence, does not equate to interaction.

## Prioritizing Co-occurring Exposures in Phenotype: Toward EWASs

Drawing inspiration from “genome-wide association studies” (GWASs), an “environment-wide association study” (or equivalently “exposome-wide association study” [EWAS]), is a straightforward method to prioritize associations between single exposures and a phenotype [17].

GWASs are a way to associate millions of genetic factors with disease or phenotype along the entirety of the genome. Due to wide accessibility of genome-scale GWAS assays that can ascertain millions of genetic variants simultaneously, human geneticists have now moved from studying a handful of genetic variants at a time to a more data-driven, comprehensive, systematic, and agnostic reporting of genetic associations and their replication in independent populations. At its simplest, GWASs are epidemiological studies that examine the association between the prevalence of genetic variants in cases (diseased) versus controls (healthy), not unlike most environmental epidemiological studies in design. Specific genetic factors are prioritized by examining what associations have a *p* value lower than a family-wise error rate (e.g., Bonferroni correction) and are replicated by testing significant associations in an independent cohort.

EWASs borrow the methods of GWAS to search for and analytically validate environmental factors associated with continuous phenotypes or cases versus controls. This type of question is different from a hypothesis-driven approach in which a single candidate or a handful of environmental factors are chosen a priori and tested individually for their association to a phenotype. Briefly, the strength of EWAS is in comprehensively testing for linear associations between each exposure and a phenotype, similar to that of GWAS studies. Instead of testing a few environmental associations at a time, EWAS evaluates multiple environmental factors. EWAS is comprehensive in that each factor measured is assessed for possible association with the target phenotype. Next, associations are systematically adjusted for multiplicity of comparisons. Finally, EWAS calls for validation of significant associations in an independent population.

In an EWAS study, *M* total exposure variables are associated with a phenotype or outcome using a linear model, iteratively or one at a time. Thus, for an environmental factor *X*_*i*_ in the list of measured factors *X*_*i*_ … *X*_*m*_, the disease state (*Y*) is modeled as a linear function of environmental factors and adjustment variables (represented by *Z*)

1 $$ Y=\alpha +{b}_i{X}_i+\gamma Z $$

*X*_*i*_ corresponds to the environmental factor, and *b*_*i*_ corresponds to the effect size of that factor (e.g., beta coefficient or odds ratio), adjusted by *Z*. The strength of association is computed by the two-sided *p* value for *b*_*i*_, which tests the “null hypothesis” that *b*_*i*_ is equal to zero. Next, to mitigate the chance for false positive discovery (type I error), a family-wise error rate, such as Bonferroni correction, is applied. The Bonferroni adjustment simply adjusts the nominal *p* value threshold by the total number of tests conducted. This adjustment guarantees the “family-wise error rate”—the probability of having one or more false positive(s). However, the threshold is conservative, and therefore, statistical power for detection is lost. An alternative to the family-wise error rate includes the false discovery rate (FDR) [18••]. The FDR is less conservative and therefore statistically more powerful than the Bonferroni correction [19]. The FDR is the estimated proportion of false discoveries made versus the number of real discoveries made for a given significance level to control for multiple hypothesis testing [18••]. The usual method of estimating the FDR is the Benjamini-Hochberg “step-down” approach; however, the approach assumes independence between tests. One way to address correlation between tests includes estimation of the FDR empirically through permutation based approaches (e.g., [20]). Further, replication in an independent dataset is sought for significant associations. We have applied EWAS to prioritize individual exposures in diabetes [21•], preterm birth [22], all-cause mortality [23], and serum lipid levels [24].

Despite comprehensively searching *M* exposures in a database with a phenotype *Y* of interest, EWAS does not explicitly find mixtures of exposures that interact to induce changes in the phenotype. EWAS does, however, identify co-occurring exposures associated with the phenotype of interest. For example, in the first EWAS for diabetes, multiple associations were identified between organochlorine compounds (e.g., PCB, organochlorine pesticides) and diabetes prevalence [21•].

**Fig. 2:**
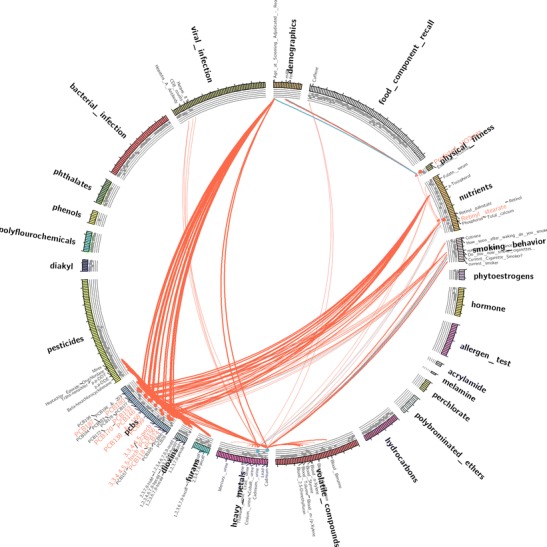
Visualization of a correlation globe focusing on exposures identified in EWAS in telomere length. Exposures identified in the EWAS procedure are seen in the *outer circle* in the *orange* (e.g., PCBs, *lower left*) or *blue* (cadmium, *bottom center*) colored font. The *orange color font* indicates a positive direction of the association in telomere length (for example, higher PCB levels were associated with longer telomere length) and a *blue color font* a negative direction in telomere length (e.g., higher cadmium levels were associated with shorter telomeres). Edges are drawn between exposures that co-occur with EWAS-identified exposures; for example, cadmium and lead are both correlated with volatile compound levels, seen in the *bottom* of the figure

In an EWAS in telomere length [25], multiple correlated PCB congeners were found in telomere length. These associations indicate that these exposures statistically co-occur in association with their respective phenotype. One can then visualize these co-occurring exposures in a focused correlation globe. For example, Fig. 2 shows a correlation globe that only shows exposures that are correlated, or co-occur, with those found in a EWAS in telomere length.

One can utilize exposure co-occurrences to increase the power of detection of exposures associated with phenotype. How? Simply put, suppose two (of the *M*) exposures measured in a cohort study co-occur 100% of the time (e.g., their correlation equals 1). Therefore, these two exposures provide redundant information, and the actual analytic number of exposures will not be *M*, but *M* − 1 (subtracting 1 of the redundant exposures that co-occur 100% of the time). Conceptually, one can estimate how redundant variables are in a dataset by computing their correlations. The difference between the number of variables measured and the number of redundant variables is known as the “effective number of exposure variables” (in the toy example above, *M* − 1 is the effective number of exposure variables). Knowing the “effective number of exposure variables,” or the number of exposures in the dataset after taking into account their co-occurrence/correlation, can increase the power of detection of exposures and mixtures by reducing the space of possible exposures to explore. As a proof-of-principle, using the NHANES, we re-estimated the effective number of variables for different categories of exposure [26••]. For example, we found many of the 38 total polychlorinated biphenyls (PCBs) measured in NHANES 1999–2004 to be redundant and estimated an effective number of PCBs to be 24, significantly less than the 38 measured. For details on how to estimate the effective number of variables to reduce the complexity of testing for exposure-phenotype associations, we direct readers to [26••].

## Emerging Analytical Methods to Identify Interactions Between Exposures Associated to Phenotype

Emerging and existing machine-learning methods will enhance detection of mixtures associated with phenotypes and disease outcomes. Typically, machine-learning approaches apply algorithms to find variables (exposures) that are predictive of an outcome (phenotype) in two steps. In the first step, an algorithm “learns” the variables that are associated with the outcome. The algorithm is then tested in an independent dataset to estimate the predictive capability or generalizability of the algorithm.

The EWAS screening method considers each environmental factor in a separate linear model one at a time or iteratively. An issue that remains includes how to generalize beyond correlated factors associated with the outcome. One solution might be to test exposures that are identified in EWAS for interaction (e.g., [27]). For example, given *L* number of exposures identified in an EWAS, an investigator may choose to test all pairwise exposures of the *L* for interaction.

To discover interactions associated with the outcome, one needs to test or model them simultaneously. However, as the reader may know, modeling all possible interactions in a linear model will lead to overfitting and non-generalizable associations. Therefore, to analytically select exposures in phenotype, one can leverage a “feature selection” algorithm, such as stepwise regression, whereby different combinations of variables are input in a model and the most predictive model is chosen while preserving model parsimony (limited the number of variables in the linear model). Classical stepwise regression is a challenge to apply due to their high variability in variable selection, ultimately reducing their prediction accuracy [28]. One alternative includes extensions to the linear model, such as regularized regression.

One class of well-known methods includes extensions to the linear model, known as “regularized” regression, such as the “least angle selection and shrinkage operator” (LASSO, [29]) or “ElasticNet” [30]. Both the LASSO and the ElasticNet avoid overfitting the model by constraining the size of coefficients (“shrinking”). One practical way of identifying interactions between *pairs* of exposure variables is to enter *all* possible pairwise exposure interactions into an algorithm such as LASSO, with a “multiplicative term” (e.g., interaction between exposure *X*_1_and exposure *X*_2_ is entered as *X*_1_ × *X*_2_), expanding the scope of Eq. 1 to the following, assuming all exposure variables are continuous (and z-standardized or mean-subtracted and divided by the standard deviation)

2 $$ Y=\alpha +{b}_1{X}_1+\ldots +{b}_m{X}_m+{a}_1{X}_1\times {X}_2+\ldots \kern0.5em +\zeta Z $$

where all exposure variables *X*_1_ through *X*_*m*_ are included in the model as “additive terms” along with all pairs of multiplicative terms for interactions between pairs of variables (e.g.,*X*_1_ × *X*_2_), resulting in a model of size *M* plus *M* choose 2. Non-zero coefficients (e.g., *a*_1_) on multiplicative terms indicate interaction. Therefore, the output of such a procedure may include a list of interacting pairs of exposures -- and their additive terms -- that are predictive of the phenotype or disease outcome after shrinkage.

Along with ElasticNet, Agier et al. demonstrate feasibility of other established and emerging methods for conducting EWAS-like analyses, including use of sparse partial least squares, “deletion/subtraction/addition” (DSA), and Graphical Unit Evolutionary Stochastic Search (GUESS) [31] in simulation studies. While promising, these methods still underperform in identifying single exposures in phenotypes as the number of correlated exposures increases, a problem that will undoubtedly influence identification of mixtures.

Another “off-the-shelf” method that may consider dependencies between exposure variables includes tree-based methods and their variants, including random forests and boosted machines. The first tree-based method, “Classification and Regression Tree” (CART; [32]) is an algorithm that selects combinations of variables, or exposures, that are predictive of the phenotype (e.g., disease) that resemble a “rule” or decision tree. A decision tree for the prediction of diabetes (versus non-diabetics in a case-control study) could resemble:

“if bisphenol A > 10 mg/dL and polychlorinated biphenyl 170 > 0.01 mg/dL then predict diabetes with probability 0.8.”

Rules or decision trees can thereby represent dependencies between multiple (greater than two) exposure variables associated with a phenotype. However, classical CART is known to have high *variance*, or their predictive capability is variable in the test step after learning in the training dataset. This occurs mainly because the algorithm is too specific or is said to overfit the training data. Newer tree-based methods, such as “random forests” aim to minimize this variance. Random forests lower variance by executing a CART-like algorithm on many randomized versions (or “bootstraps”) of the training dataset and then selecting a prediction decision tree that produces an average prediction that results from the trees built on the multiple randomized versions of the data. Usually, these trees are deep and represent decision rules that are complex.

Lampa and colleagues applied another variant of a tree-based approach to identify multiple exposures of a mixture associated with a phenotype called “boosting” [33•]. Boosted trees, unlike random forests, grow many small (e.g., two to three exposures) trees that each aim to reduce the prediction error. However, because random forests and boosted trees harness predictive capability of multiple trees (often hundreds and thousands of trees), additional analytical steps must be taken to ascertain how pairs of exposure variables are dependent or interact. This is done through estimation of Friedman and Popescu’s *H* statistic [34]. In essence, the statistic estimates how much variance can be explained by interaction between pairs of exposure versus their additive contributions by the boosted tree algorithm and can be modified to ascertain interactions between multiple exposures. Lampa and colleagues tested this method to identify mixtures in 27 correlated exposures (e.g., PCBs, bisphenol A, etc.) in 1000 participants that were predictive of serum bilirubin. The investigators tentatively found an interaction between PCB-127 and bisphenol A in predicting serum bilirubin concentrations. Specifically, they found that an increase in the biomarker levels of both of these analytes results in a greater than additive increase in serum bilirubin levels.

## Power, Replication, and Interpretation of Identified Mixtures

Practical considerations that can also be acknowledged to identify mixtures in phenotypes include (1) adequate statistical power or, equivalently, sample size, (2) interpretation of mixture-phenotype associations, and (3) replication of mixture-phenotype associations. First, it is well known that large sample sizes are required to find pairwise and higher order interactions. Conceptually, detecting mixtures is a type of “stratified” analysis. For example, suppose an investigator is attempting to identify an effect of a mixture consisting of two binary-valued exposures (*E*_1_ and *E*_2_) in a phenotype *Y*. Since *E*_1_ and *E*_2_ are binary valued, there are four possible configurations of the two exposures of the mixture. Inferring a mixture or interaction effect between *E*_1_ and *E*_2_ on *Y* requires data on all four strata of *E*_1_ and *E*_2_, increasing the sample size requirement to detect stratum-specific associations in *Y*. As described above, considering more than two-way interactions between exposures in a putative mixture will consist of immense number of combinations, increasing the power burden. We claim that with current cohort sample sizes (i.e., hundreds to low thousands), it may be impossible to reproducibly find interactions between more than a few exposures in a phenotype.

Second, how does one interpret a mixture? Interpretation of pairwise variables is often conveyed through stratified analyses, whereby risk or correlations in a phenotype (*Y*) of one exposure (e.g., *X*_1_) are displayed for different levels of another exposure (e.g., *X*_2_) [35, 36]. In a simple regression setting, this can be written as the following

3 $$ Y=\alpha +{\beta}_1{X}_1+{\beta}_2{X}_2+{\beta}_3{X}_1\times {X}_2 $$

However, testing a three-way interaction between a mixture of three exposures (*X*_1_, *X*_2_, and *X*_3_) is analytically more complex. For example, in a simple regression setting, the three-way interaction model is written as follows

4 $$ Y=\alpha +{\beta}_1{X}_1+{\beta}_2{X}_2+{\beta}_3{X}_3+{\beta}_4{X}_1\times {X}_2+{\beta}_5{X}_1\times {X}_3+{\beta}_6{X}_2\times {X}_3+{\beta}_7{X}_1\times {X}_3+{\beta}_8{X}_1\times {X}_2\times {X}_3 $$

This model is much more difficult to interpret than one that contains only pairwise interaction terms (e.g., do all interaction coefficients, *β*_5_ , *β*_6,_ *β*_7_ , *β*_8_, have to be non-zero to infer a mixture?—the answer to this question is not an easy one to answer). Third, and relatedly, replicating a single exposure-phenotype association can be reduced to a simple heuristic: the effect sizes must be concordant in independent cohorts or datasets. But how does one replicate a set of exposures associated with a phenotype? There are numerous coefficients to verify consistency when attempting to replicating associations between mixtures in phenotype, increasing the possibility of a false negative or positive finding. Model choice, or what mixture configurations (e.g., two-way, three-way, or *N*-way give example) are input into the model, will influence associations and inference [37].

## Conclusions

It is hypothesized that environmental factors act in concert, or as mixtures, to induce changes in phenotype and risk for disease. The sheer number of possible combinations of exposures makes the identification of mixture-phenotype associations an analytic challenge. Specifically, identifying mixtures (i.e., interaction between exposures in a phenotype) is resource intensive and requires large sample sizes and power. Second, interpretation and replication of synergistic relationships between exposures associated to a phenotype are not as straightforward as interpreting single exposure-phenotype associations.

However, going forward in the coming high-throughput exposome era, we claim that methods such as EWAS, may help prioritize single agents which can then be tested systematically to identify mixtures. Secondly, narrowing down the potential mixtures by searching for naturally co-occurring exposures in databases such as NHANES may also enhance the search for prevalent mixtures associated with phenotypes (e.g., examples in Fig. 1 and Table 1); however, this will come at the cost of not identifying rare mixtures that contain rare exposures. Third, there is promise in extending statistical machine learning methods, such as regularized regression and tree-based methods, to identify dependent exposures. We suggest that more research effort be devoted in developing new analytic methods. In fact, as of this writing, the National Institute of Environmental Health Sciences (NIEHS) has issued a request for application (i.e., https://grants.nih.gov/grants/guide/rfa-files/RFA-ES-17-001.html) and sponsored a workshop [9•] to promote the development of new methods to identify mixtures. Ultimately, high-throughput measurement of exposure indicators (e.g., the exposome) will enable environmental health researchers to dissect comprehensive environmental burden of disease. However, new methods and larger datasets must be built to address the vast complexity of a large number of potential mixtures that could exist in phenotypic variability.

## References

[1] Rappaport SM, Smith MT (2010). Environment and disease risks. Science.

[2] Wild CP (2012). The exposome: from concept to utility. Int J Epidemiol.

[3] Athersuch TJ (2012). The role of metabolomics in characterizing the human exposome. Bioanalysis.

[4] Carpenter DO, Arcaro K, Spink DC (2002). Understanding the human health effects of chemical mixtures. Environ Health Perspect.

[5] Carlin DJ, Rider CV, Woychik R, Birnbaum LS (2013). Unraveling the health effects of environmental mixtures: an NIEHS priority. Environ Health Perspect.

[6] Hill AB (1965). The environment and disease: association or causation?. Proc R Soc Med.

[7] Ioannidis JPA (2016). Exposure-wide epidemiology: revisiting Bradford Hill. Stat Med.

[8] Fedak KM, Bernal A, Capshaw ZA, Gross S (2015). Applying the Bradford Hill criteria in the 21st century: how data integration has changed causal inference in molecular epidemiology. Emerg Themes Epidemiol.

[9] Taylor KW, Joubert BR, Braun JM, Dilworth C, Gennings C, Hauser R, Heindel JJ, Rider CV, Webster TF, Carlin DJ (2016). Statistical approaches for assessing health effects of environmental chemical mixtures in epidemiology: lessons from an innovative workshop. Environ Health Perspect.

[10] • Manrai AK, Cui Y, Bushel PR, et al. (2017) Informatics and data analytics to support exposome-based discovery for public health. Annu Rev Public Health 38. **This review presents new approaches to ascertain exposome-phenotype associations inspired by a decade of genome-phenotype association investigations.** 10.1146/annurev-publhealth-082516-012737 PMC5774331 28068484

[11] Braun JM, Gennings C, Hauser R, Webster TF (2016). What can epidemiological studies tell us about the impact of chemical mixtures on human health. Environ Health Perspect.

[12] • Patel CJ, Manrai AK (2015) Development of exposome globes to map out environment-wide associations. Pac. Symp. Biocomput. **An easy-to-implement method and visualization technique to identify clusters of exposures through correlation.** PMC4299925 25592584

[13] Butte AJ, Kohane IS (2000). Mutual information relevance networks: functional genomic clustering using pairwise entropy measurements. Pac Symp Biocomput.

[14] Krzywinski M, Schein J, Birol I, Connors J, Gascoyne R, Horsman D, Jones SJ, Marra MA (2009). Circos: an information aesthetic for comparative genomics. Genome Res.

[15] Smith GD, Lawlor DA, Harbord R, Timpson N, Day I, Ebrahim S (2007). Clustered environments and randomized genes: a fundamental distinction between conventional and genetic epidemiology. PLoS Med.

[16] Ioannidis JPA, Loy EY, Poulton R, Chia KS (2009). Researching genetic versus nongenetic determinants of disease: a comparison and proposed unification. Sci Transl Med.

[17] Patel CJ, Ioannidis JPA (2014). Studying the elusive environment in large scale. J Am Med Assoc.

[18] Benjamini Y, Hochberg Y (1995). Controlling the false discovery rate: a practical and powerful approach to multiple testing. Journal-Royal Statistical Society Series B.

[19] Noble WS (2009). How does multiple testing correction work?. Nat Biotechnol.

[20] Storey JD, Tibshirani R (2003). Statistical significance for genomewide studies. Proc Natl Acad Sci U S A.

[21] Patel CJ, Bhattacharya J, Butte AJ (2010). An environment-wide association study (EWAS) on type 2 diabetes mellitus. PLoS One.

[22] Patel CJ, Yang T, Hu Z (2014). Investigation of maternal environmental exposures in association with self-reported preterm birth. Reprod Toxicol.

[23] Patel CJ, Rehkopf DH, Leppert JT, Bortz WM, Cullen MR, Chertow GM, Ioannidis JPA (2013). Systematic evaluation of environmental and behavioural factors associated with all-cause mortality in the United States National Health and Nutrition Examination Survey. Int J Epidemiol.

[24] Patel CJ, Cullen MR, Ioannidis JPA, Butte AJ (2012). Systematic evaluation of environmental factors: persistent pollutants and nutrients correlated with serum lipid levels. Int J Epidemiol.

[25] Patel CJ, Manrai AK, Corona E, Kohane IS (2016). Systematic correlation of environmental exposure and physiological and self-reported behaviour factors with leukocyte telomere length. Int J Epidemiol.

[26] Patel CJ, Ioannidis JPA (2014). Placing epidemiological results in the context of multiplicity and typical correlations of exposures. J Epidemiol Community Health.

[27] Patel CJ, Ioannidis JPA, Cullen MR, Rehkopf DH (2015). Systematic assessment of the correlations of household income with infectious, biochemical, physiological, and environmental factors in the United States, 1999-2006. Am J Epidemiol.

[28] Vittinghoff E, Glidden D, Shiboski S, McCulloch C (2005). Regression methods in biostatistics: linear, logistic, survival, and repeated measures models.

[29] Tibshirani R (1996). Regression shrinkage and selection via the lasso. J R Stat Soc Series B Stat Methodol.

[30] Zou H, Hastie T (2005). Regularization and variable selection via the elastic net. J R Stat Soc Series B Stat Methodol.

[31] Agier L, Portengen L, Chadeau-Hyam M (2016). A systematic comparison of linear regression-based statistical methods to assess exposome-health associations. Environ Health Perspect.

[32] Breiman L, Friedman J, Stone CJ, Olshen RA. Classification and regression trees: CRC Press; 1984.

[33] Lampa E, Lind L, Lind PM, Bornefalk-Hermansson A (2014). The identification of complex interactions in epidemiology and toxicology: a simulation study of boosted regression trees. Environ Health.

[34] Friedman JH, Popescu BE (2008). Predictive learning via rule ensembles. Ann Appl Stat.

[35] Patel CJ, Chen R, Kodama K, Ioannidis JPA, Butte AJ (2013). Systematic identification of interaction effects between genome- and environment-wide associations in type 2 diabetes mellitus. Hum Genet.

[36] Patel CJ (2016). Analytical complexity in detection of gene variant-by-environment exposure interactions in high-throughput genomic and exposomic research. Curr Environ Health Rep.

[37] Patel CJ, Burford B, Ioannidis JPA (2015). Assessment of vibration of effects due to model specification can demonstrate the instability of observational associations. J Clin Epidemiol.

